# Clinical and economic outcomes of pharmacological stress tests in patients with a history of COVID‐19

**DOI:** 10.1002/clc.24008

**Published:** 2023-03-23

**Authors:** Hicham Skali, David Walker, Jeanette Jiang, Giridharan Gurumoorthy, Kalatu Davies, Tomomi Kimura

**Affiliations:** ^1^ Brigham and Women's Hospital Boston Massachusetts USA; ^2^ Astellas Pharma Global Development Inc. Northbrook Illinois USA; ^3^ Astellas Pharma US Inc. Northbrook Illinois USA

**Keywords:** COVID‐19, pharmacological stress test, regadenoson, reversal agent, United States

## Abstract

**Background:**

Despite millions of COVID‐19 cases in the United States, it remains unknown whether a history of COVID‐19 infection impacts the safety of pharmacologic myocardial perfusion imaging stress testing (pharmacologic MPI).

**Hypothesis:**

The aim of this study was to assess if a prior COVID‐19 infection was associated with a higher risk of complications during and following pharmacologic MPI testing.

**Methods:**

This retrospective cohort analysis included 179 803 adults (≥18 years) from the PharMetrics® Plus claims database who underwent pharmacologic MPI between March 1, 2020 and February 28, 2021. Patients with a history of COVID‐19 infection (COVID‐19 group) were compared with propensity‐score matched no‐COVID‐19 history group for reversal agent use, 30‐day resource use, and post‐MPI cardiac events/procedures.

**Results:**

The most commonly used stress agent was regadenoson (91.7%). The COVID‐19 group (*n* = 6372; 3.5%) had slightly higher: reversal agent use (difference 1.13% [95% confidence interval [CI]: 0.33, 1.92]), all‐cause costs (difference USD $128 [95% CI: $73–$181]), and office visits (81.5% vs. 77.0%) than the no‐COVID‐19 group. Prior COVID‐19 infection did not appear to impact subsequent cardiac events/procedures.

**Conclusions:**

COVID‐19 history was associated with slightly higher reversal agent use, all‐cause costs, and office visits after pharmacologic MPI; however, the differences were not clinically meaningful. Concerns for use of stress agents in patients with prior COVID‐19 do not appear to be warranted.

## Graphical abstract

In a retrospective cohort analysis of 179 803 adults from a large claims database, prior COVID‐19 infection did not impact reversal agent use or resource costs associated with pharmacologic myocardial perfusion imaging (MPI) stress testing in a clinically meaningful way. Overall, reductions in the use of pharmacologic MPI during early 2020 and some regional differences in reversal agent use were noted.

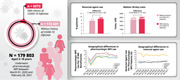

**Abbreviations**
AMI: acute myocardial infarction
CI: confidence interval
CL: confidence limit
HR: hazard ratio
OR: odds ratio
pharmacologic MPI: pharmacologic myocardial perfusion imaging stress testing

## 1 INTRODUCTION

Over the last 2.5 years, severe acute respiratory syndrome coronavirus 2 (SARS‐CoV‐2), or COVID‐19, infections affected several million individuals with a broad range of complications and outcomes, ranging from short‐lived effects with no or mild symptoms, to prolonged hospitalizations, severe complications, or death.^1^, ^2^ In addition to their baseline risk, COVID‐19 survivors may have a heightened risk of cardiac complications and may be referred for pharmacologic myocardial perfusion imaging stress tests (pharmacologic MPI) for symptoms related, or not, to their prior COVID‐19 infection.^3^, ^4^, ^5^, ^6^, ^7^, ^8^ Depending on the local setting, COVID‐19 incidence, and availability, the use of pharmacologic MPI may be recommended instead of an exercise stress test to reduce droplet exposure risk and thus avoid the potential spread of COVID‐19.^9^ Although lab closures in response to local and national recommendations early in the pandemic resulted in reduced cardiac diagnostic testing procedures, volumes of procedures in the United States and Canada in 2021 slightly surpassed prepandemic levels.^10^ However, it remains unknown whether a prior COVID‐19 infection impacts the safety of pharmacologic MPI.

Guidelines recommend regadenoson as the preferred coronary vasodilator agent for pharmacologic MPI during the pandemic^9^ due to its shorter infusion time (10 seconds) compared with several minutes for adenosine and dipyridamole, which helps to minimize contact time between health professionals and patients.^11^, ^12^, ^13^ In the event of serious or intolerable adverse effects during pharmacologic MPI, reversal agents such as caffeine and the adenosine receptor antagonist (aminophylline) can be administered to reverse the effects of the vasodilator agent.^14^, ^15^, ^16^, ^17^ In the regadenoson phase 3 trials of 2015 patients undergoing pharmacologic MPI (median [range] age: 66 [26–93] years; primarily White males), 3% of patients receiving regadenoson (*n* = 46/1337) and 2% of those receiving adenosine (*n* = 12/678) received reversal agents.^11^, ^18^ Recently, Hasnie et al. reported that 1 out of 15 patients (6.7%) who recently recovered from COVID‐19 used aminophylline.^19^

The aim of this study was to assess if a prior COVID‐19 infection was associated with a higher risk of complications during and following pharmacologic MPI testing. Specifically, the effect of a prior history of COVID‐19 infection on reversal agent use, subsequent healthcare visits, all‐cause costs, and cardiac events/procedures following pharmacologic MPI was examined.

## 2 METHODS

### 2.1 Study design and data source

This retrospective cohort analysis used the IQVIA PharMetrics® Plus claims database, a longitudinal health‐plan database of medical and pharmacy claims, including 200 million enrollees from US national and subnational health plans and self‐insured employer groups. The study was conducted in compliance with all national requirements for non‐interventional studies using deidentified data. The data from IQVIA PharMetrics® Plus is permitted to be in research and publications; informed consent, ethics committee approval, and Institutional Review Board approval were not necessary for this study.

### 2.2 Study population

The study population consisted of patients aged ≥18 years who underwent pharmacologic MPI between March 1, 2020 and February 28, 2021 (the available data set at the time of study design). The index date was defined as the first pharmacologic MPI date for the patient. Patients were required to have at least 1 year of preindex continuous enrollment (a maximum gap of 1 month was allowed to minimize patient attrition) to identify any comorbidities and COVID‐19 diagnosis before pharmacologic MPI. Patients without valid birth‐year and sex information were not included in the cohort. Patients with a history of a heart transplant, those with the use of >1 type of pharmacological stress agent on the index date, and those who underwent a pharmacologic MPI procedure within 30 days before the index date were also excluded.

Patients were divided into two groups based on the presence of a history of COVID‐19 infection, determined by the use of the International Classification of Diseases 10th revision diagnosis code U07.1. To assess the impact of the proximity of COVID‐19 infection to pharmacologic MPI, patients with a history of COVID‐19 infection were classified by active COVID‐19 infection (i.e., the initial diagnosis of COVID‐19 infection ≤14 days before the index date) or by recovered COVID‐19 infection (i.e., initial diagnosis >14 days prior). Additionally, patients with severe COVID‐19 infection were defined as those who required hospitalization; other patients were defined as nonsevere patients.

### 2.3 Outcome definitions

Reversal agent use was defined by procedure codes or National Drug Code on the index date. Among those requiring reversal agent use, a prespecified diagnosis was sought as a potential indication of reversal agent use.^14^, ^15^, ^16^, ^17^ Thirty‐day all‐cause costs after pharmacologic MPI (in US dollars [USD]) were assessed among patients excluding the Medicare Supplemental population because this database does not capture the Medicare part of the payment. Thirty‐day healthcare visits were also assessed. Healthcare visits included hospitalization, emergency room visits, outpatient hospital visits, and office visits.

Cardiac events and procedures occurring within 90 days after pharmacologic MPI were defined as cardiac angiography/catheterization without revascularization, revascularization without acute myocardial infarction (AMI), and AMI.^20^

### 2.4 Statistical analyses

Patients with a history of COVID‐19 infection were matched to those without a history of COVID‐19 infection using propensity scores to balance out prognostic characteristics and comorbidities. Propensity scores were derived from a logistic model with a dependent variable of COVID‐19 history at index date, and independent variables during the preindex period including age category, index month, and presence of each of 17 Charlson comorbidity index conditions. Gender, geographic divisions, index month before or after December 2020, and payer type (Medicare supplemental or not) were forced to match. Another propensity score was estimated using a multinomial logistic model to compare severe COVID‐19 history versus no COVID‐19 history and nonsevere COVID‐19 history versus no COVID‐19 history.

The rate of reversal agent use and proportion with each type of healthcare visit were compared between the matched groups. The 95% confidence intervals (CIs) for the difference in frequencies were estimated based on *t* distribution, and odds ratios (ORs) were estimated using logistic models. The impact on all‐cause costs was estimated as median differences between the matched pairs and 95% confidence limits (CLs) for the median difference were estimated by distribution‐free CLs for quantiles.

Factors associated with reversal agent use were explored using multivariate logistic regression models including the history of COVID‐19 infection, age category (<65 vs. 65+), gender, pharmacological stress agent, place of service (office vs. hospital outpatient vs. others), index year, geographic division, and individual Charlson comorbidity index conditions as covariates. The ORs and the respective 95% CIs were estimated. The cumulative incidence in 90 days of cardiac events or procedures was calculated using Kaplan–Meier methods and compared between matched patients with a history of COVID‐19 infection and patients without a history of COVID‐19 infection. Hazard ratios (HRs) were estimated using a Cox proportional hazard model. Note that this analysis was performed in the subgroup of patients who were enrolled by December 31, 2020 to include ≥90 days of follow‐up in the database.

Chronological trends in pharmacologic MPI use during the study period were assessed by taking potential changes in the size of the database itself into account (e.g., by leaving insurance programs). The monthly pharmacologic MPI rate/10 000 beneficiaries were estimated by dividing the number of monthly indexes pharmacologic MPI from March 2020 to February 2021 by the monthly total number of beneficiaries in the database and multiplying by 10 000. The monthly reversal agent use rate by geographic region was estimated among pharmacologic MPI patients with known geographic regions. Reversal agent use rate (%) was estimated per geographical region and was calculated as a proportion of pharmacologic MPI with reversal‐agent use among total pharmacologic MPI in each region.

The statistical analysis plan was developed and iteratively revised by the authors. Statistical analyses were performed using SAS software, Version 9.04.

## 3 RESULTS

### 3.1 Patients

In total, 179 803 patients with complete data underwent pharmacologic MPI and were included in the study (Supporting Information: Figure S1). Age ranged from 52 to 69 years, and 64% (*N* = 115 668) of patients were aged <65 years (Table 1). A slightly greater proportion of patients were men (53.9%). The most commonly used stress agent during pharmacologic MPI was regadenoson (91.7%). There was a total of 6372 (3.5%) patients with a history of COVID‐19 infection, which was classified as nonsevere in 4607 (72.3%) patients and severe in 1765 (27.7%) patients. The proportion of patients with a history of COVID‐19 increased from 0% in March 2020 to 24.6% in February 2021 in this cohort. Patients with a prior COVID‐19 infection tended to be younger and were more likely to have chronic pulmonary disease than patients without prior COVID‐19. Other baseline demographics and clinical characteristics were otherwise generally similar between the two groups.

**Table 1:** Baseline characteristics of patients who underwent pharmacologic MPI, by prior history of COVID‐19 (overall and propensity‐matched cohorts)

Parameter	Total cohort (before matching)		Matched cohort	
	Without history of COVID‐19 infection (*n* = 173 431)	With history of COVID‐19 infection (*n* = 6372)	Without history of COVID‐19 infection (*n* = 19 101)	With history of COVID‐19 infection (*n* = 6367)
Age, median^a^ (Q1–Q3), years	62 (56–69)	59 (52–64)	58 (52–64)	59 (52–64)
Men, *n* (%)	93 689 (54.0)	3186 (50.0)	9543 (50.0)	3181 (50.0)
MPI^b^, *n* (%)				
SPECT	156 454 (90.2)	5778 (90.7)	17 332 (90.7)	5776 (90.7)
PET	17 045 (9.8)	597 (9.4)	1777 (9.3)	594 (9.3)
Stress agent, *n* (%)				
Regadenoson	159 009 (91.7)	5831 (91.5)	17 579 (92.0)	5826 (91.5)
Dipyridamole	7683 (4.4)	309 (4.8)	832 (4.4)	309 (4.9)
Adenosine	6739 (3.9)	232 (3.6)	690 (3.6)	232 (3.6)
Comorbidities, *n* (%)				
Chronic pulmonary disease	40 559 (23.4)	1811 (28.4)^c^	5224 (27.3)	1810 (28.4)
Diabetes without chronic complication	32 801 (18.9)	1375 (21.6)	4190 (21.9)	1372 (21.5)
Diabetes with chronic complication	28 168 (16.2)	1127 (17.7)	3033 (15.9)	1125 (17.7)
Congestive heart failure	28 181 (16.2)	1141 (17.9)	3053 (16.0)	1141 (17.9)
Renal disease	22 572 (13.0)	868 (13.6)	2242 (11.7)	867 (13.6)
Myocardial infarction	17 143 (9.9)	699 (11.0)	1851 (9.7)	699 (11.0)
The geographic region of the United States, *n* (%)				
Midwest	42 356 (24.4)	1698 (26.6)	5079 (26.6)	1693 (26.6)
Northeast	22 001 (12.7)	730 (11.5)	2190 (11.5)	730 (11.5)
South	84 579 (48.8)	3355 (52.7)	10 065 (52.7)	3355 (52.7)
West	24 006 (13.8)	584 (9.2)^c^	1752 (9.2)	584 (9.2)
Unknown	489 (0.3)	5 (0.1)	15 (0.1)	5 (0.1)
Index month, *n* (%)				
March 2020	13 097 (7.6)	0^c^	0	0
April 2020	7850 (4.5)	14 (0.2)^c^	46 (0.2)	14 (0.2)
May 2020	12 536 (7.2)	105 (1.6)^c^	308 (1.6)	105 (1.6)
June 2020	16 916 (9.8)	166 (2.6)^c^	491 (2.6)	166 (2.6)
July 2020	17 132 (9.9)	252 (4.0)^c^	780 (4.1)	252 (4.0)
August 2020	16 332 (9.4)	374 (5.9)^c^	1146 (6.0)	374 (5.9)
September 2020	16 692 (9.6)	522 (8.2)	1531 (8.0)	522 (8.2)
October 2020	16 971 (9.8)	563 (8.8)	1654 (8.7)	563 (8.8)
November 2020	14 725 (8.5)	661 (10.4)	1942 (10.2)	661 (10.4)
December 2020	14 756 (8.5)	950 (14.9)^c^	2923 (15.3)	950 (14.9)
January 2021	13 244 (7.6)	1200 (18.8)^c^	3549 (18.6)	1198 (18.8)
February 2021	13 180 (7.6)	1565 (24.6)^c^	4731 (24.8)	1562 (24.5)

Abbreviations: PET, positron emission tomography; pharmacologic MPI, pharmacologic myocardial perfusion imaging stress testing; Q, quartile; SPECT, single‐photon emission computed tomography.

^a^ In the IQVIA PharMetrics® Plus database, 85+ years of age is summarized as “age 85.”

^b^ Some patients had records for both SPECT and PET on the same day.

^c^ Standardized mean difference >0.1.

### 3.2 Reversal agent use

In total, before matching, reversal agents were used in 13 890 patients (7.7%). Reversal agents used were aminophylline (98.0%) and caffeine (2.0%); theophylline was not used. The most common diagnoses given on the date of reversal agent use were chest pain (54.6%) and arrhythmia (13.8%).

Reversal agent use was higher in patients with a history of COVID‐19 infection than in those without a history of COVID‐19 infection (8.8% vs. 7.7%; difference 1.1% [95% CI: 0.4, 1.8]). Among patients aged <65 years, reversal agent use was higher among those with a COVID‐19 history than those without a COVID‐19 history (8.9% vs. 7.3%); while among patients aged ≥65 years, reversal agent use was similar in those with or without a history of COVID‐19 infection (8.4% vs. 8.3%). In general, reversal agent use appeared to be higher in women (9.4%) than in men (6.3%).

**Figure 1:**
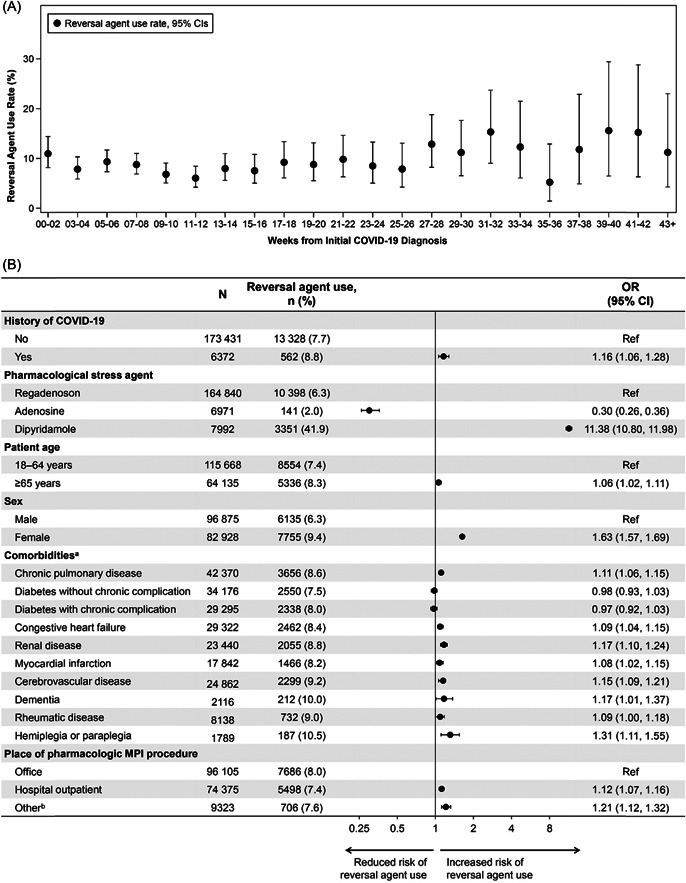
(A) Reversal agent used according to time since initial COVID‐19 diagnosis and (B) multivariable model of predictors of reversal agent use during pharmacologic MPI (total cohort). ^a^A reference exposure was not used for comorbidities OR calculations. ^b^Includes inpatient visits and emergency room visits. CI, confidence interval; pharmacologic MPI, pharmacologic myocardial perfusion imaging stress testing; OR, odds ratio; Ref, reference.

Among patients with a history of COVID‐19 infection, reversal agent use was lower in patients who had experienced severe COVID‐19 than in those who had experienced nonsevere COVID‐19 (8.4% vs. 9.0%). Use of reversal agents was higher among patients with recent COVID‐19 infection (≤2 weeks between COVID‐19 diagnosis and pharmacologic MPI; 11.0%) compared to those with pharmacologic MPI greater than 2 weeks after COVID‐19 diagnosis (8.7%). There was no significant change over time with longer time since the COVID‐19 diagnosis (Figure 1A).

In a multivariate model, factors associated with reversal agent use included: a history of COVID‐19 infection, age ≥65 years, female sex, use of dipyridamole as a pharmacological stress agent, comorbidities (myocardial infarction, congestive heart failure, cerebrovascular disease, dementia, chronic pulmonary disease, hemiplegia or paraplegia, and renal disease), and MPI place of service in an outpatient hospital or other settings (vs. physicians' office) (Figure 1B).

Table 1 includes propensity‐matched cohorts and shows that baseline characteristics were well‐balanced. Reversal agent use was slightly higher in patients with COVID‐19 history, but the difference was minimal (difference 1.13% [95% CI: 0.33, 1.92]; OR: 1.16 [95% CI: 1.05, 1.28]; Table 2). When stratified by COVID‐19 severity, differences remained statistically significant for nonsevere COVID‐19 (difference 1.72% [95% CI: 0.79, 2.65]; OR: 1.26 [95% CI: 1.12, 1.42]), but not for severe COVID‐19 (difference 0.61% [95% CI: −0.88, 2.09]; OR: 1.08 [95% CI: 0.89, 1.32]).

**Table 2:** Use of reversal agents during pharmacologic MPI, according to prior history of COVID‐19 (matched cohort).

	History of COVID‐19 infection (overall)		History of nonsevere COVID‐19 infection		History of severe COVID‐19 infection	
	No (*n* = 19 101)	Yes (*n* = 6367)	No^a^ (*n* = 13 821)	Yes (*n* = 4607)	No^a^ (*n* = 5289)	Yes (*n* = 1763)
Reversal agent use, *n* (%)	1471 (7.7)	562 (8.8)	1004 (7.3)	414 (9.0)	412 (7.8)	148 (8.4)
Difference (95% CI), %	1.13 (0.33, 1.92)		1.72 (0.79, 2.65)		0.61 (–0.88, 2.09)	
Odds ratio (95% CI)	1.16 (1.05, 1.28)		1.26 (1.12, 1.42)		1.08 (0.89, 1.32)	

Abbreviations: CI, confidence interval; pharmacologic MPI, pharmacologic myocardial perfusion imaging stress testing.

^a^ No history of COVID‐19 infection.

### 3.3 All‐cause costs and healthcare visits

All‐cause costs were minimally higher among those with versus those without a history of COVID‐19 infection (Table 3). The median cost of care in the 30 days following a pharmacologic MPI was $128 higher per patient among those with a prior COVID‐19 infection. The largest difference was observed for office visits being more frequent among those with a prior COVID‐19 infection. There was no difference in hospitalization rates, but rates for emergency‐room visits, outpatient hospital visits, and physician office visits were significantly higher in patients with COVID‐19 history than without prior COVID‐19.

**Table 3:** Resource use in patients who underwent pharmacologic MPI according to prior history of COVID‐19 (matched cohort).

Parameter	Without a history of COVID‐19 infection	With a history of COVID‐19 infection
30‐Day costs, USD		
Patients^a^, *n*	18 384	6128
Median (Q1–Q3)	3470 (1812–7479)	3607 (1954–7833)
Difference (95% CI), USD	128 (73, 181)^b^	
30‐Day healthcare visit rate (%)		
Patients, *n*	19 101	6367
Hospitalization, *n* (%)	793 (4.2)	274 (4.3)
Emergency‐room visit, *n* (%)	1211 (6.3)	478 (7.5)^b^
Outpatient hospital visit, *n* (%)	8351 (43.7)	2906 (45.6)^b^
Office visit, *n* (%)	14 708 (77.0)	5188 (81.5)^b^

Abbreviations: CI, confidence interval; pharmacologic MPI, pharmacologic myocardial perfusion imaging stress testing; Q, quartile; SD, standard deviation; USD, United States Dollar.

^a^ Patients with Medicare supplemental insurance (*n* = 15 309) were excluded from the cost analysis because the Medicare portion of the payment was not available in the source database.

^b^ Statistically significant differences between the groups.

### 3.4 Cardiac outcomes after pharmacologic MPI

**Figure 2:**
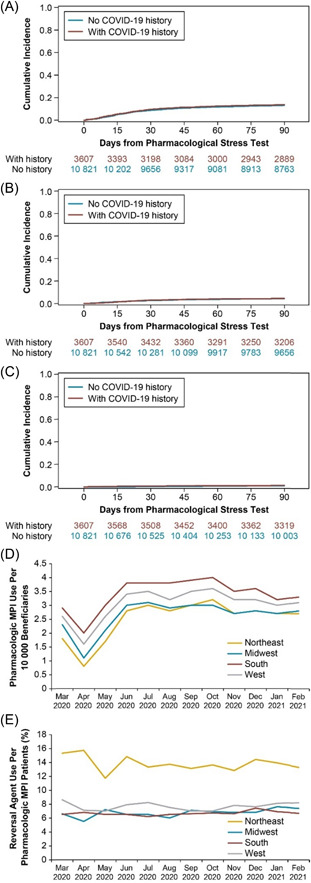
Cumulative incidence of (A) angiography/catheterization, (B) revascularization, or (C) acute myocardial infarction over 90 days from pharmacologic MPI in the matched cohort; geographical differences in (D) pharmacologic MPI use and (E) reversal agent use. (D) The number of pharmacologic MPI per month was counted and divided by the total number of beneficiaries in the database and multiplied by 10 000 to obtain pharmacologic MPI per 10 000 beneficiaries for each month. (E) Monthly reversal agent use per index MPI by geographic region. The rate of patients with missing geographic regions was excluded from this analysis. Pharmacologic MPI, pharmacologic myocardial perfusion imaging stress testing.

Among patients enrolled by December 2020, there was no difference in cumulative incidence of cardiac events or procedures between patients with a history of COVID‐19 infection and those without during the 90‐day post‐pharmacologic MPI period (Figure 2A–C). There was no difference in HRs between the two groups in cardiac angiography without revascularization (HR: 1.07; 95% CI: 0.97, 1.19), revascularization without AMI (HR: 1.01; 95% CI: 0.85, 1.22), or AMI (HR: 1.31; 95% CI: 0.89, 1.91).

### 3.5 Chronological trends in pharmacologic MPI and geographical variations in reversal agent use

Overall rates of pharmacologic MPI use from March 2020 to February 2021 were similar between the Northeast, Midwest, South, and West regions (Figure 2D). Slightly lower pharmacologic MPI rates were observed between March and May 2020 (2.5/10 000 beneficiaries), with a dip in April 2020 (1.5/10 000 beneficiaries) but a recovery in June 2020 (3.4/10 000 beneficiaries). The reversal agent use rate against total pharmacologic MPI was also slightly lower in April 2020 (7.2%) but rather stable during the study period (7.2%–8.2%).

Reversal agent use generally remained unchanged over the entire period (Figure 2E). The rate of reversal agent use was higher from March 2020 to February 2021 in the Northeast region than in the Midwest, South, and West regions. Overall, the rate was highest in the Northeast region (13.7% [3111/22 731]), whereas the lowest rate was observed in the South (6.6% [5846/87 934]). Regadenoson was used in over 90% of pharmacologic MPI procedures.

## 4 DISCUSSION

Our study shows that patients with a history of COVID‐19 infection undergoing pharmacologic MPI had a slightly higher reversal agent use than those without prior COVID‐19 history (8.8% vs. 7.7%, respectively). Among patients who underwent pharmacologic MPI, subsequent resource use and costs were minimally impacted by COVID‐19 history (difference USD $128; 95% CI $73–$181). History of COVID‐19 infection did not appear to impact cardiac events or procedures such as angiography/catheterization, revascularization, or AMI.

In a much smaller single‐center study of 15 patients undergoing SPECT MPI (4 exercises, 11 pharmacologic [all regadenoson]) after recovery from COVID‐19‐related hospitalization, none experienced any serious adverse events during or after stress testing, with only one patient requiring a reversal agent (aminophylline).^19^ In our study, reversal agent use was slightly lower in patients who had experienced severe versus nonsevere COVID‐19 (8.4% vs. 9.0%) and in patients who had recovered from COVID‐19 versus those with active COVID‐19 infection (8.7% vs. 11.0%). However, the nonadjusted rates may still be confounded and these differences are likely not clinically significant.

Some regional differences in reversal agent use were noted. This is potentially due to geographical variations in drug availability or prescribing practice. Locations may have different reversal agents of choice and some may routinely use reversal agents as a prophylactic.^14^, ^21^

Similar to our observation, other studies have reported a decline in the use of MPI in general at the beginning of the COVID‐19 pandemic, followed by a return to at least prepandemic levels.^10^, ^22^, ^23^, ^24^ Reductions in the use of pharmacologic MPI during early 2020 correspond to the issuing of local and national recommendations to preserve personal protective equipment and reduce transmission of the SARS‐CoV‐2 virus between patients and medical staff at the peak of the pandemic in the United States, by deprioritizing elective procedures that may have included cardiovascular imaging tests.^25^, ^26^, ^27^, ^28^, ^29^ While there may be a hint of increased rates of pharmacologic MPI use after the initial significant drop in April 2020, it is unknown whether this is due to catch‐up of postponed cases, an increased number of patients requiring MPI because of cardiac sequelae of COVID‐19, or increased use of pharmacologic MPI over exercise stress tests as initially recommended by the American Society of Nuclear Cardiology, International Atomic Energy Agency, and Society of Nuclear Medicine and Molecular Imaging COVID‐19 information statement.^9^ It is notable that 3.5% of patients in this study had a history of COVID‐19 infection, in contrast to the approximately 28 million cumulative cases of COVID‐19 (8%–9% of the population) in the United States by February 2021.^30^ This is likely a consequence of the studied sample, as IQVIA PharMetrics® Plus is a national claims database of mostly privately insured patients and only a few older Medicare patients.

While the relatively large number of included patients is a notable strength of this study, there remain a few limitations: the unavoidable retrospective design; the possible COVID‐19 history misclassification; the reduced generalizability due to the relatively small proportion of patients older than age 65; and the lack of data relative to racial or ethnic representation. Finally, after 2 years from the end of the study period (February 28, 2021), we may now have more patients with a history of COVID‐19 infection. Such patients may or may not be similar to our study population, for example, in terms of cardiac risk by different variants. Therefore, our findings may not be generalizable to the current patient population.

The results of this study could help alleviate concerns regarding the safety of pharmacological stress agents in patients with a history of COVID‐19 infection. However, there remains a need for additional studies to better understand the effect of a prior COVID‐19 infection on the performance and results of exercise or pharmacologic stress testing.

## 5 CONCLUSIONS

In a large claims database of patients undergoing pharmacologic MPI, a prior COVID‐19 infection was associated with slightly higher reversal agent use, all‐cause costs, and office visits; however, the differences were not clinically meaningful. Concerns for use of stress agents in patients with prior COVID‐19 do not appear to be warranted.

## AUTHOR CONTRIBUTIONS

David Walker and Tomomi Kimura conceived and developed the study. Hicham Skali, David Walker, Jeanette Jiang, and Tomomi Kimura provided acquisition, analysis, or interpretation of data. All authors critically revised each draft of the manuscript and reviewed and approved the final version.

## CONFLICTS OF INTEREST STATEMENT

Hicham Skali has received consulting fees from Astellas Pharma and research support from ABT Associates. David Walker is an employee of Astellas Pharma. Jeanette Jiang is an employee of Astellas Pharma. Giridharan Gurumoorthy is an employee of Astellas Pharma. Kalatu Davies is a former employee of Astellas Pharma. Tomomi Kimura is an employee of Astellas Pharma.

## Supporting information

Supporting Information.

## Data Availability

Researchers may request access to anonymized participant‐level data, trial‐level data, and protocols from Astellas‐sponsored clinical trials at www.clinicalstudydatarequest.com. For the Astellas criteria on data sharing see: https://clinicalstudydatarequest.com/Study-Sponsors/Study-Sponsors-Astellas.aspx.
