# Supplementary material for: Clinical and economic outcomes of pharmacological stress tests in patients with a history of COVID‐19
Source: Clin Cardiol. 2023 Mar 23;46(5):558–66. doi: 10.1002/clc.24008 (PMC10189073; doi:10.1002/clc.24008)
Supplement: Supplementary file 1 — Supporting Information. [file CLC-46-558-s001.docx]

**Supporting Information**

**Supplementary Figure S1.** Patient disposition


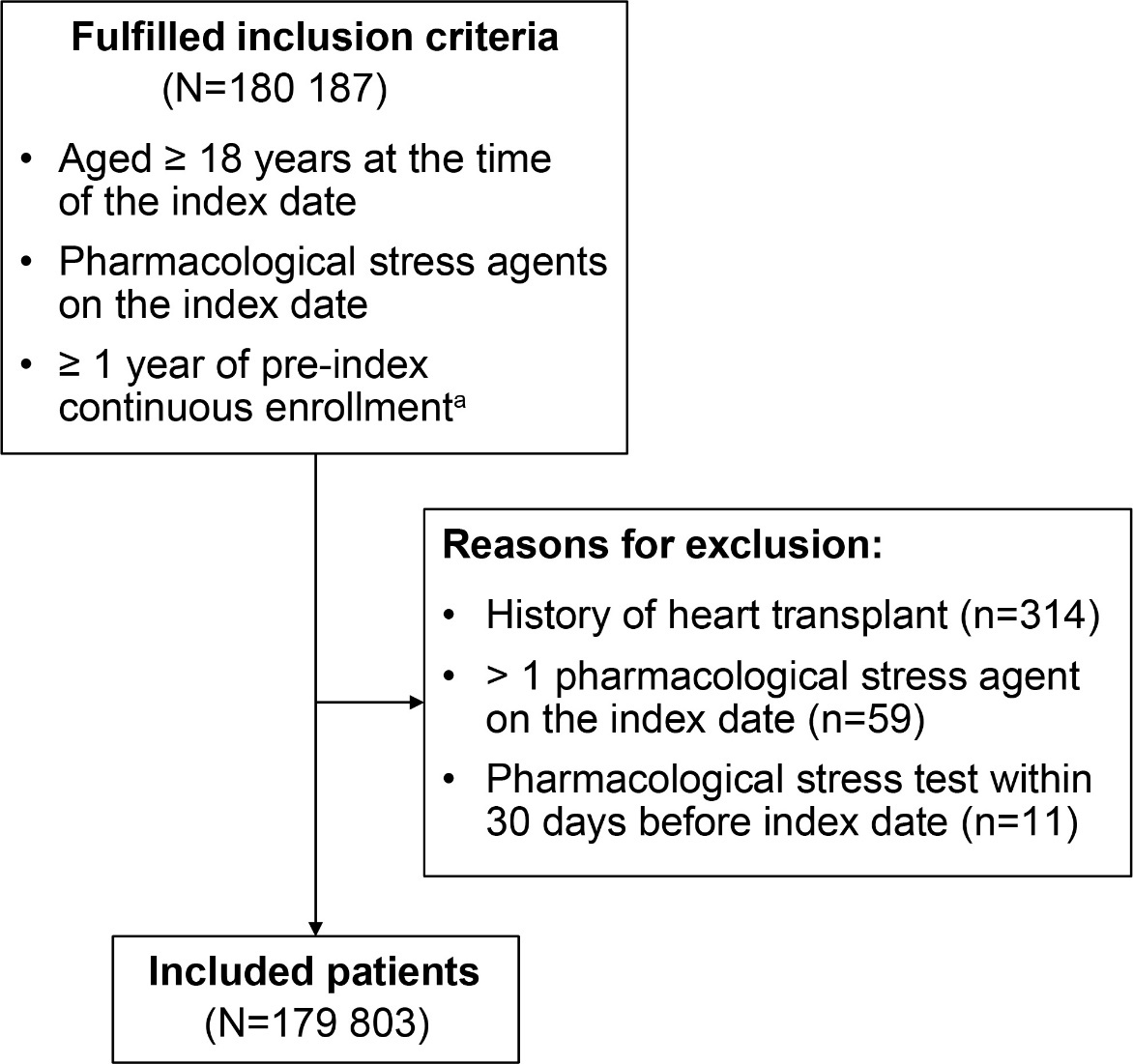


^a^Maximum one-month gap allowed.
